# Divergent responses to thermogenic stimuli in BAT and subcutaneous adipose tissue from *interleukin 18* and *interleukin 18 receptor 1*-deficient mice

**DOI:** 10.1038/srep17977

**Published:** 2015-12-10

**Authors:** Patricia Pazos, Luis Lima, Sulay Tovar, David González-Touceda, Carlos Diéguez, María C. García

**Affiliations:** 1 Department of Physiology/Research Center of Molecular Medicine and Chronic Diseases (CIMUS). University of Santiago de Compostela; 2 Instituto de Investigación Sanitaria de Santiago de Compostela, Santiago de Compostela, Spain; 3 CIBER Fisiopatología Obesidad y Nutrición (CB06/03), Instituto de Salud Carlos III (ISCIII), Ministerio de Economía y Competitividad (MINECO), Spain

## Abstract

Brown and beige adipocytes recruitment in brown (BAT) or white adipose tissue, mainly in the inguinal fat pad (iWAT), meet the need for temperature adaptation in cold-exposure conditions and protect against obesity in face of hypercaloric diets. Using *interleukin18* (*Il18)* and *Il18 receptor 1*- knockout (*Il18r1*-KO) mice, this study aimed to investigate the role of IL18 signaling in BAT and iWAT activation and thermogenesis under both stimuli. *Il18*-KO, extremely dietary obesity-prone as previously described, failed to develop diet-induced thermogenesis as assessed by BAT and iWAT *Ucp1* mRNA levels. Overweight when fed standard chow but not HFD, HFD-fed *Il18r1*-KO mice exhibited increased iWAT *Ucp1* gene expression. Energy expenditure was reduced in pre-obese *Il18r1*-KO mice and restored upon HFD-challenge. Cold exposure lead to similar results; *Il18r1*-KO mice were protected against acute body temperature drop, displaying a more brown-like structure, alternative macrophage activation and thermogenic gene expression in iWAT than WT controls. Opposite effects were observed in *Il18*-KO mice. Thus, *Il18* and *Il18r1* genetic ablation disparate effects on energy homeostasis are likely mediated by divergent BAT responses to thermogenic stimuli as well as iWAT browning. These results suggest that a more complex receptor-signaling system mediates the IL18 adipose-tissue specific effects in energy expenditure.

Obesity-induced inflammation has important effects on morphological and functional properties of the adipocyte^1^. Interleukin (IL) 18 is a IL1 family of ligands and receptors, member, primarily associated with acute- and chronic-inflammation. Classically known as an interferon (IFN)-γ co-stimulator with IL12, a new modulatory role of IL18 in lipid and glucose metabolism has recently emerged^2^,^3^ Bacterial and inflammatory stimuli induces IL18 production by immune and non-immune cells in several metabolic organs/tissues, such as brain, liver, skeletal muscle, and specially adipose tissue (AT)^4^.

The IL18 receptor (IL18R) is composed of a ligand binding (IL18R1) and a signal-transducing chain or accessory protein (IL18RAP), both essential for MYD88 and IL1 receptor-associated kinase 1 (IRAK1) recruitment, subsequent translocation to the nucleus of nuclear factor (NF)-KB and pro-inflammatory gene transcription^5^,^6^. IL18 also triggers energy metabolism signaling pathways such as those of signal transducer and activator of transcription 3 (STAT3), as well as mitogen-activated protein, phosphoinositide-3 and AMP-activated protein kinases (MAPK, PI3K and AMPK)^7^,^8^. Central and peripheral IL18 activity seem to be tightly regulated by naturally occurring inhibitors: its high affinity soluble binding protein (IL18BP) and two splice variants of its receptor subunits, claimed to act as decoy receptors^9^,^10^,^11^. A second non-competitive ligand of IL18R1 with potent anti-inflammatory action, namely IL37, also exits^12^,^13^. However, the complex IL37/ IL18R1 does not bind IL18RAP. Instead, it recruits an inhibitory co-receptor, the IL1R family member SIGIRR (also known as IL1R8), to engage downstream signaling events^14^,^15^.

Within last decade, transgenic mice studies have been essential in determining the potential role of IL18 signaling in energy homeostasis^2^,^8^,^16^. Former studies of Netea^2^ and Zorrilla^16^ *et al.* showed that *Il18* null mice develop mature onset obesity not only due to hyperphagia and hypoactivity, but also disturbances in peripheral nutrient metabolism^17^. Thus, *Il18* deficiency decreased insulin sensitivity and increased fuel-efficiency, whereas central and/or peripheral IL18 administration reverses these effects. Additionally, *Il18r1* absence and *Il18bp* overexpression in mice led to insulin resistance, hyperglycemia and obesity^2^. Conversely, skeletal muscle *Il18*-overexpression induces resistance to dietary obesity in mice through activation of AMPK signaling and lipid oxidation in this tissue^8^. Interestingly IL37 is also expressed in subcutaneous and visceral AT of human donors^18^. Though no IL37 mouse homolog has been cloned yet, mice with transgenic overexpression of human IL37 are resistant to high-fat diet (HFD), showed reduced AT macrophages, and upregulation of mitochondrial energy metabolism related genes in this tissue^18^.

AT is nowadays regarded as a diverse dynamic organ^19^,^20^. Brown AT (BAT) mitochondria inefficiently oxidizes fatty acids from BAT or white AT (WAT) lipolysis in response to cold or high-caloric diets, thereby dissipating energy as heat in an attempt to maintain body temperature and energy balance^21^,^22^,^23^. A main molecular mediator of this energy sparing mechanism is uncopling protein 1 (Ucp1)^24^,^25^. Data gleaned recently have shown that WAT depots develop brown fat features in response to thermogenic stimuli^26^,^27^. This browning process, more prominent in the inguinal subcutaneous fat (iWAT) than in other visceral fat depot, has attracted considerable interest due to its possible beneficial role on total energy metabolism^28^.

Over the last years it has became clear that skeletal muscle releases endocrine signals and metabolites such as irisin, IL6 or BAIBA with ability to induce a white to brown shift in adipocytes^29^,^30^. However, despite the relevant role of IL18/IL18R system in overall energy homeostasis, muscle and AT metabolism, and its reported interaction with different fat browning related signaling pathways, the specific involvement of this cytokine and its receptor on adipocyte plasticity remains unexplored. The present study aims to clarify the potential adaptive function of IL18 signaling on adipose tissue metabolism under different thermogenic stimuli: HFD and cold. For this purpose, we exposed *Il18* and *Il18r1* deficient mice to HFD and analyzed their metabolic phenotype. The impact of cold-exposure on BAT function and thermal responses was also analyzed, as were its effects on brown fat-like gene program in iWAT.

## Results

### Differential responses of weight gain, food intake, BAT and iWAT *Ucp1* gene expression to long-term HFD in *Il18r1* and *Il18*-KO mice

**Figure 1:**
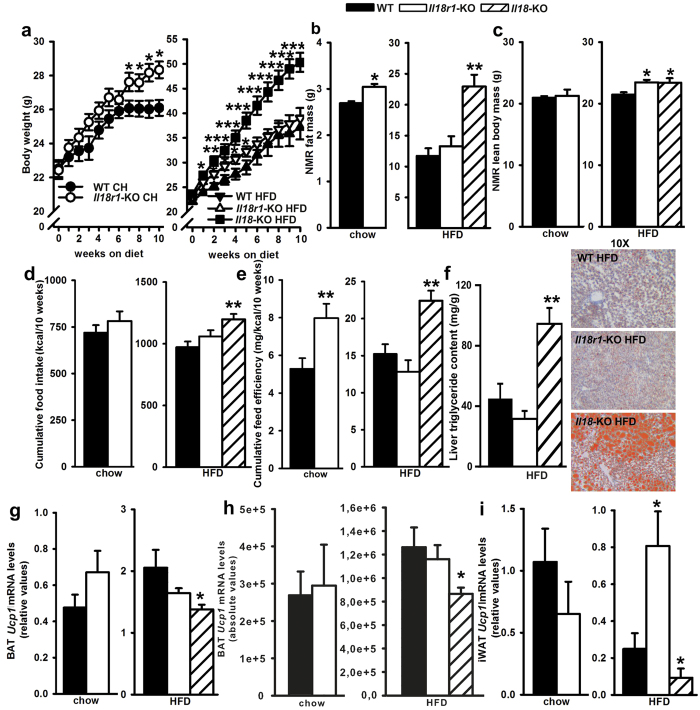
Divergent responses to dietary obesity in *Il18r1* and *Il18*-KO mice. (**a**) Body weight gain of WT, *Il18r1* and *Il18*-KO mice fed a regular chow (CH) or a 60% high-fat diet (HFD) for 10 weeks (n = 8–13). (**b,c**) Fat and lean body mass as determined by NMR at the end of the study (n = 8–13). (**d**) Cumulative food intake per animal and cage (n = 4–6) and (**e**) feed efficiency across the 10-weeks feeding period (n = 8–13). (**f**) Hepatic triglyceride content and representative images (10X magnification) of liver sections from WT, *Il18r1* and *Il18*-KO HFD mice stained with oil-red to determine the accumulation of neutral fat (left panel). (**g**–**i**) *Ucp1* mRNA levels in BAT and iWAT (n = 7). Data are expressed as mean ± SEM. *P < 0.05, **P < 0.01 and ***P < 0.001 versus respective WT mice fed the same diet by two-way ANOVA for repeated measurements ((**a**) CH: genotype F (1, 23) = 7.088, time F (10, 230) = 31.65 and genotype X time interaction F (10, 230) = 1.915; P < 0.05, P < 0.0001 and P < 0.05; HFD: genotype F (2, 31) = 12.85, time F (10, 310) = 173.8 and genotype X time interaction F (20, 310) = 2.463; P < 0.001 for all), and two-tailed Student´s t-test or one-way ANOVA.

Firstly, we explored changes in body weight, food intake, feed efficiency and body composition in *Il18r1* and *Il18*-KO mice consuming a 60% HFD for 10 weeks. The metabolic performance of both strains was previously compared^2^,^16^ and found to be similar, thus only a group of *Il18r1*-KO mice fed a standard 4% fat diet as internal control was included. In this study, chow-reared *Il18r1*-KO mice exhibited significantly greater body weight than WT mice, with significant differences beginning already at 15 weeks of age (Fig. 1a). At the end of the experiment, *Il18r1*-KO mice were 8.6% heavier than WT controls and had significantly increased total fat but not lean body mass as determined by NMR analysis (Fig. 1b,c). Intra-abdominal dissected fat weight was 20% greater in *Il18r1*-KO compared to WT mice, whereas no effect was observed in iWAT mass (supplementary Fig. S1a). Relative fat content was, however, only slightly increased in *Il18r1*-KO mice at this age (supplementary Fig. S1b). Of note, detailed observation of body composition in mature (24 weeks old) mice revealed that though *Il18r1*-KO had similar body weight than *Il18* deficient mice, they exhibited lower total and gonadal AT mass, smaller iWAT adipocytes cell size as well as lower BAT lipid accumulation (supplementary Fig. S2).

Lack of *Il18* but not *Il18r1* in mice led to increased susceptibility to dietary obesity (Fig. 1a). Despite higher weight gain in *Il18r1*-KO mice during the initial four weeks on HFD, at the end of the study their body weight as well as total and abdominal adiposity were similar to those of WT mice (Fig. 1a,c, and supplementary Fig. S1a). On the contrary, final fat mass in *Il18*-KO was 80.4% greater than in WT mice (Fig. 1b), due to heightened abdominal and iWAT fat accumulation (1.6 to 2.2-fold increase vs WT controls; supplementary Fig. S1a). Absolute lean body mass was also slightly increased in animals deficient in the ligand or its receptor (Fig. 1c). Concordant with overweight and adiposity, leptin levels were greater in *Il18* but not in *Il18r1*-KO compared with WT animals (Table 1). Additionally, reflecting a disturbed glucose metabolism, HFD-fed *Il18*-KO mice showed increased non-fasted glucose levels, and yet unexpectedly higher adiponectin levels than WT mice (Table 1).

*Il18* deficiency in mice has been associated with increased food consumption and fuel efficiency on both low- and high-fat diets as well as reduced energy expenditure^2^,^16^. In the present work, *Il18r1*-KO and WT mice ate similar amounts of calories from each diet (Fig. 1d), but only exhibited greater feed efficiency when maintained on regular chow (Fig. 1e). Consistent with previous published results^16^, *Il18*-KO HFD mice were hyperphagic and gained 1.7-fold more body weight than WT mice per unit energy consumed (Fig. 1e).

Feed efficiency differences depending on the dietary fat amount suggested that *Il18r1*-KO were less obesity-prone than *Il18*-null mice, through either divergence in substrate utilization and/or energy expenditure. In support of the first hypothesis, subtle differences in whole-fat oxidation indices have been reported between these two transgenic mice models^8^,^16^,^17^. Hence, while mature *Il18*-KO mice consuming HFD ad libitum have increased respiratory exchange ratio (RER), this effect is only evident in mature *Il18r1*-KO mice after fasting and refeeding. In the present study and reflecting a failure in liver lipid metabolism, hematoxylin-eosin staining revealed increased hepatic steatosis (Fig. 1f), paralleled by higher triglyceride content (Fig. 1g) as well as markedly decreased serum triglyceride levels in HFD-fed mice with absence of the ligand but not of the receptor (Table 1). Additionally, we examined BAT metabolism, known to contribute to plasma triglyceride clearance by oxidizing free fatty acids for heat production^26^,^31^, and the alternative pathway dissipating energy through increased thermogenic activity by iWAT browning. *Ucp1* gene expression in BAT and iWAT samples from the diet intervention study were assessed (Fig. 1g–i). HFD-fed *Il18*-KO displayed increased BAT weights (Supplementary Table S1) as well as lower BAT and iWAT *Ucp1* mRNA absolute or relative levels (per μg of mRNA) than WT mice raised on the same diet, suggesting reduced thermogenic capacity as part of the obesogenic mechanism. Of note, the HFD-induced down-regulation of iWAT *Ucp1* mRNA levels was blunted in *Il18r1*-KO mice, which exhibited higher *Ucp1* mRNA content in this fat depot than that of WT controls. No differences were found in BAT weight and either total or relative *Ucp1* gene expression between WT and *Il18r1*-KO mice irrespective of diet.

### Lack of *Il18r1* in young mice results in decreased energy expenditure on standard chow but not on HFD

**Figure 2:**
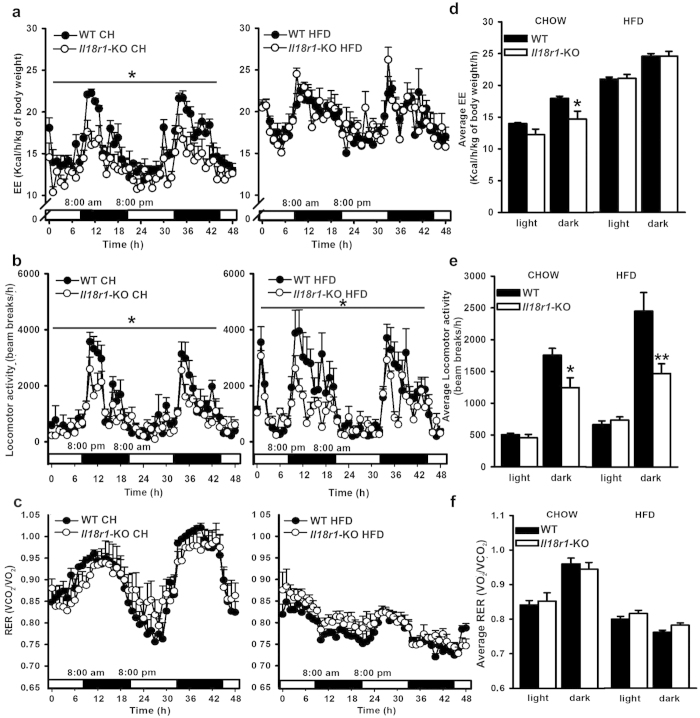
High fat diet challenge increased EE in *Il18r1*-KO mice. (**a**–**c**) Energy expenditure (EE), total locomotor activity and respiratory exchange ratio (RER) were measured at room temperature in 8-weeks old WT and *Il18r1*-KO mice fed regular chow (CH, left panel) and then switched to high fat diet (HFD, right panel) for consecutive periods of 48 h. Black horizontal bars depict the dark period in a 12:12 light-dark cycle. (**d**–**f**) Average EE, locomotor activity and RER values over the light and dark period before (left panel) and after HFD challenge (right panel). Data are expressed as mean ± SEM. (n = 6). *P < 0.05 and **P < 0.01 versus respective WT mice fed the same diet by two-way ANOVA for repeated measurements ((**a**) CH: genotype F (1, 10) = 5.087, time F (48, 480) = 13.70 and genotype X time interaction F (48, 480) = 1.706; P < 0.05, P < 0.0001and P < 0.01; (b) CH: genotype F (1, 10) = 5.753, time F (48, 480) = 12.32 and genotype X time interaction F (48, 480) = 1.655; P < 0.05, P < 0.0001 and P < 0.01) and two-tailed Student´s t-test.

Next, we analyzed the effect of diet composition on the different components of daily energy expenditure (EE) in young pre-obese *Il18r1*-KO mice. For this purpose, mice were initially fed standard chow for two days and subsequently HFD-challenged for another 48 hours. Indirect calorimetry revealed that, under standard diet, EE in *Il18r1*-KO mice was significantly lower in relation to WT mice (Fig. 2a). The decrease was mainly observed during night time (Fig. 2d), with a slight but not significant reduction in average EE values during day time (WT CH-13.985 ± 0.125 vs. *Il18r1*-KO CH-12.236 ± 0.88 Kcal/kg/h; P = 0.077). This nocturnal reduction in EE was related to a diminished physical activity in these animals (Fig. 2b), as demonstrated by 29% lower dark-phase total locomotor activity in *Il18r1*-KO compared to WT mice (Fig. 2e). Once switched to HFD, *Il18r1*-KO mice raised their EE values to WT mice levels (Fig. 2a,d), without changes in nocturnal activity (Fig. 2b,e). There were no significant RER differences between genotypes whether fed chow or HFD (Fig. 2c,f). These data suggest that *Il18r1*-KO mice might display an increased diet-induced thermogenesis that obscures the reductive effect of activity on total EE.

### Divergent responses to acute cold-exposure in *Il18r1* and *Il18*-KO mice

**Figure 3:**
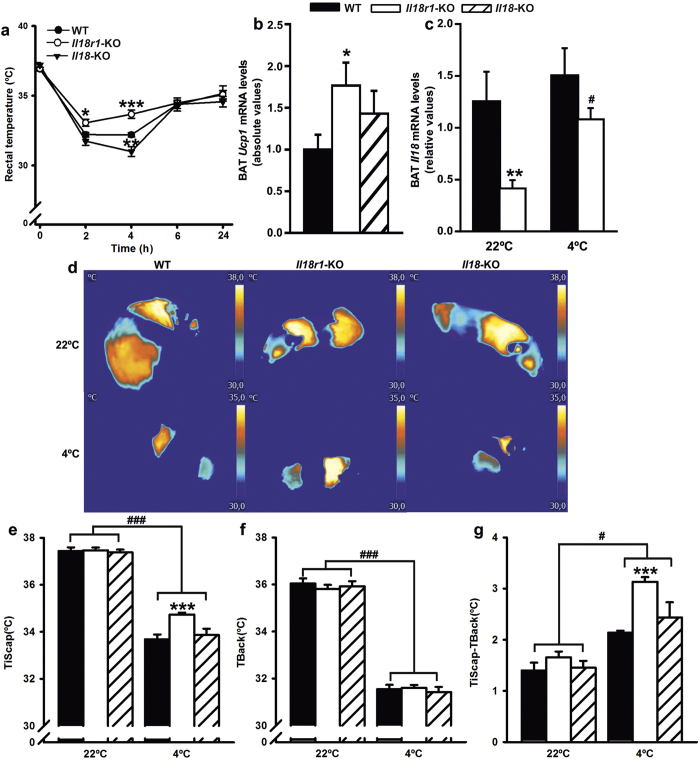
Divergent responses to short time cold exposure of rectal and BAT temperature, and BAT *Ucp1* mRNA content in *Il18r1* and *Il18*-KO mice. (**a**) Body temperature at different times after cold exposure (4 °C) in WT, *Il18r1* and *Il18*-KO mice (n = 8–12). (**b,c**) BAT *Ucp1* and *Il18* mRNA levels after 4 h of transfer to a cold room (n = 5–6). (**d**) Representative infrared thermal images with quantification of interscapular (TiScap) and lumbar back (TBack) skin temperature at 22 °C and after 1 h at 4 °C (n = 5–8). (**e**,**f**) Average changes in TiScap and TBack, respectively and (**g**) difference TiScap-TBack that reveals the heat that is locally generated under the interscapular skin (i.e., potentially of iBAT origin) (n = 5–8). Data are expressed as mean ± SEM, *P < 0.05 and ***P < 0.001 versus WT control at the same temperature and ^#^P < 0.05 and ^###^P < 0.001 versus animals from the same genotype at room temperature by two-tailed Student’s t-test and one way ANOVA or two-way ANOVA for repeated measurements ((**a**) genotype F (2, 25) = 5.101, time F (4, 100) = 219.5 and genotype X time interaction F (25, 100) = 3.061; P < 0.05 and P < 0.0001; (**d–f**) TiScap: genotype F (2, 16) = 7.441 and genotype X temperature F (2, 16) = 6.314, P < 0.01 for both; TBack: genotype F (2, 16) = 0.1543 and genotype X temperature F (2, 16) = 0.4903, P > 0.05 for both; TiScap-TiBack: genotype F (2, 16) = 10.36, temperature F (1, 16) = 75.90 and genotype X temperature F (2, 16) = 7.113; P  <  0.01, P < 0.001 and P < 0.01, respectively).

To further dissect whether lack of *Il18* or its receptor results in altered whole-body thermogenic capacity, we measured body temperature before and during 24 h cold-exposure (Fig. 3a). Basal rectal temperatures were similar in WT, *Il18r1* and *Il18*-KO mice (WT = 36.9 °C ± 0.1, *Il18r1*-KO = 37.2 °C ± 0.2 and *Il18*-KO = 36.9 °C ± 0.1). In contrast, after transfer to a cold-environment, significant genotype and time effects as well as genotype-time interactions on rectal temperature values were observed. Thus, over the first 4 hours at 4 °C, whereas *Il18*-KO were less cold-tolerant than WT mice, *Il18r1*-null mice exhibited better ability to maintain their body temperature (Fig. 3a), and increased total BAT *Ucp1* mRNA expression despite similar tissue weight in all three genotypes (Fig. 3b and supplementary Table. S1). BAT *Il18* mRNA analysis revealed that *Il18r1* deficiency in mice is associated with decreased gene expression levels of its ligand cytokine, which were restored to WT mice values after 4 hours at 4 °C (Fig. 3c). By 24 hours, rectal temperature returned to ≈2 °C below its basal values in all mice groups and the genotype effect disappeared. Similar responses were observed when either heterozygous *Il18r1*-KO and WT littermate mice or *Il18r1*-KO and WT mice generated from mating F1 offspring from *Il18r1* heterozygote breeders were challenged to acute cold (supplementary Fig. S3a-b).

The effect of short-term cold-exposure on BAT activation was also assessed by non-invasively infrared-thermography^32^ in *Il18r1* and *Il18*-KO mice. For this purpose, animals kept at 22 °C were transferred to a cold-room for one hour, and temperatures of areas surrounding interscapular BAT (TiScap) and lumbar back skin (TBack) were measured (Fig. 3e–g). We found significant genotype effect and genotype and temperature interactions on TiScap but not TBack. Subsequent post-hoc tests revealed significantly lower cold-challenge induced reductions of TiScap in *Il18r1*-KO than in WT mice (Fig. 3e,f).

As expected^33^, reflecting BAT activation and thermogenesis, the interscapular skin laying directly above BAT cooled down less than the lumbar back skin. Temperature differences between both regions were clearly seen on TiScap-TBack (Fig. 3g), which was markedly increased in *Il18r1*-KO in relation to WT mice.

### Lack of *Il18r1* and *Il18* in mice differentially modulates thermogenic program in iWAT and BAT after long-term cold-exposure

**Figure 4:**
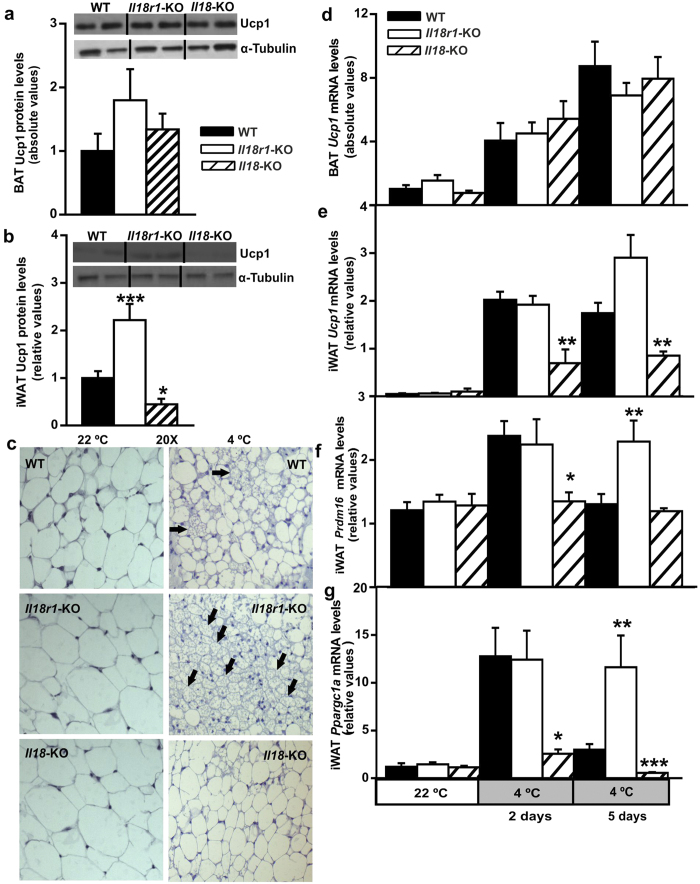
Divergent responses to long-term cold exposure of Ucp1 protein content, and BAT markers gene expression in BAT and iWAT from *Il18r1* and *Il18*-KO mice. (**a,b**) Ucp1 protein levels in BAT and iWAT, and (**c**) representative pictures (20X magnification) of iWAT histology from WT, *Il18r1* and *Il18*-KO mice maintained at room temperature or after 5 days of cold exposure. Black arrows depict multilocular adipocytes presence. (**d**) *Ucp1* mRNA content in BAT and (**e–g**) *Ucp1*, *Prdm16* and *Ppargc1a* mRNA levels in iWAT of WT, *Il18r1* and *Il18*-KO mice acclimated to either 22 °C or 4 °C for 2 and 5 days (n = 5). For absolute Ucp1 and *Ucp1* protein and mRNA levels in BAT mean value of WT mice at 4 °C (**a**) or 22 °C (**d**) were set to 1.0 and the other values expressed relative to this. Data are expressed as mean ± SEM. *P < 0.05, and ***P < 0.001 versus WT controls at the same temperature by one way ANOVA.

To gain further insight on the effect of IL18 system on BAT and iWAT thermogenic capacity and nonshivering-thermogenesis acquisition^24^, we first examined Ucp1 protein levels per depot after 5-days of cold-exposure in *Il18r1* and *Il18*-KO mice. Whereas BAT weight and total Ucp1 protein content were unaffected by lack of the ligand or the receptor, iWAT protein synthesis per μg of tissue was found to be enhanced in *Il18r1* and reduced in *Il18*-KO mice in comparison to WT controls (supplementary Table. S1 and Fig. 4a,b). Consistently, abundant patches of multilocular brown-like adipocytes found in iWAT from *Il18r1*-KO mice, were totally absent in *Il18*-null animals tissue sections (Fig. 4c). No effect of lack of *Il18* and *Il18r1* were found in total BAT *Ucp1* mRNA content (Fig. 4d). Conversely, as early as the second day of cold-challenge and onwards, significantly reduced uncoupling gene expression was observed in iWAT from *Il18*-KO but not in *Il18r1*-KO mice (Fig. 4e). To determine possible mediators of the IL18 system effects on cold-induced browning process in iWAT, we evaluated the expression of two major BAT markers: PR domain containing 16 (*Prdm16)* and peroxisome proliferative activated receptor, gamma, coactivator 1 alpha (*Ppargc1a)*. *Il18r1* deficiency upregulated both *Prdm16* and *Ppargc1a* mRNA levels in 5-days cold-exposed mice (Fig. 4f,g), whereas its gene expression was lower in *Il18*-KO mice in comparison to WT mice after two days at 4 °C (Fig. 4f).

**Figure 5:**
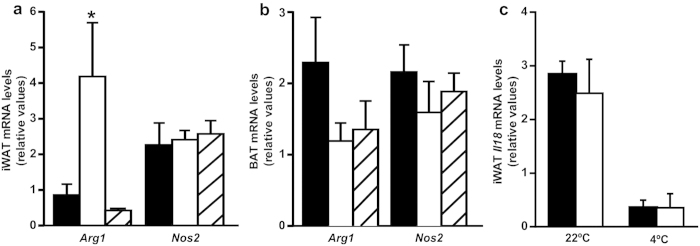
Cold exposure acutely promotes higher alternative polarization of infiltrated macrophages in iWAT from *Il18r1*-KO mice. (**a,b**) Alternative and classic macrophage polarization (*Arg1* and *Nos2*) in iWAT and BAT, and (**c**) *Il18* mRNA levels in iWAT from WT, *Il18r1* and *Il18*-KO mice after 4 h of transfer to a cold room (n = 5–6). Data are expressed as mean ± SEM, *P < 0.05 versus WT control at the same temperature by one-way ANOVA.

Cold exposure rapidly promotes alternative activation of adipose tissue macrophages (ATMs), which secrete catecholamines and anti-inflammatory cytokines to induce lipolysis as well as BAT and iWAT thermogenic gene expression^34^,^35^. To further clarify whether the effect of *Il18r1* deficiency in iWAT activation was mediated by alternative macrophage polarization, we measured the gene expression of the alternative or classic activation markers arginase (Arg1) and inducible nitric oxide synthase (Nos2)^34^ in iWAT and BAT samples of 4-hours cold exposed *Il18r1* and *Il18*-KO and WT mice (Fig. 5a,b). Higher *Arg1* mRNA levels were found in iWAT but not in BAT from *Il18r1* in relation to WT mice, but not in *Il18*-KO mice. No differences were observed in *Nos2* mRNA content in both tissues between genotypes. Similar results were obtained in *Il18r1*-KO and WT mice generated from mating homozygous F1 offspring from *Il18r1* heterozygote breeders (supplementary Fig. S3c). This specific effect of *Il18r1* deficiency in macrophages activation seemed not to be mediated by changes in locally produced Il18, as iWAT *Il18* mRNA levels were equally down-regulated by acute cold exposure in WT and *Il18r1*-KO mice (Fig. 5c).

### Chronic central IL18 administration induces BAT-like morphology in iWAT from *Il18r1*-KO mice

**Figure 6:**
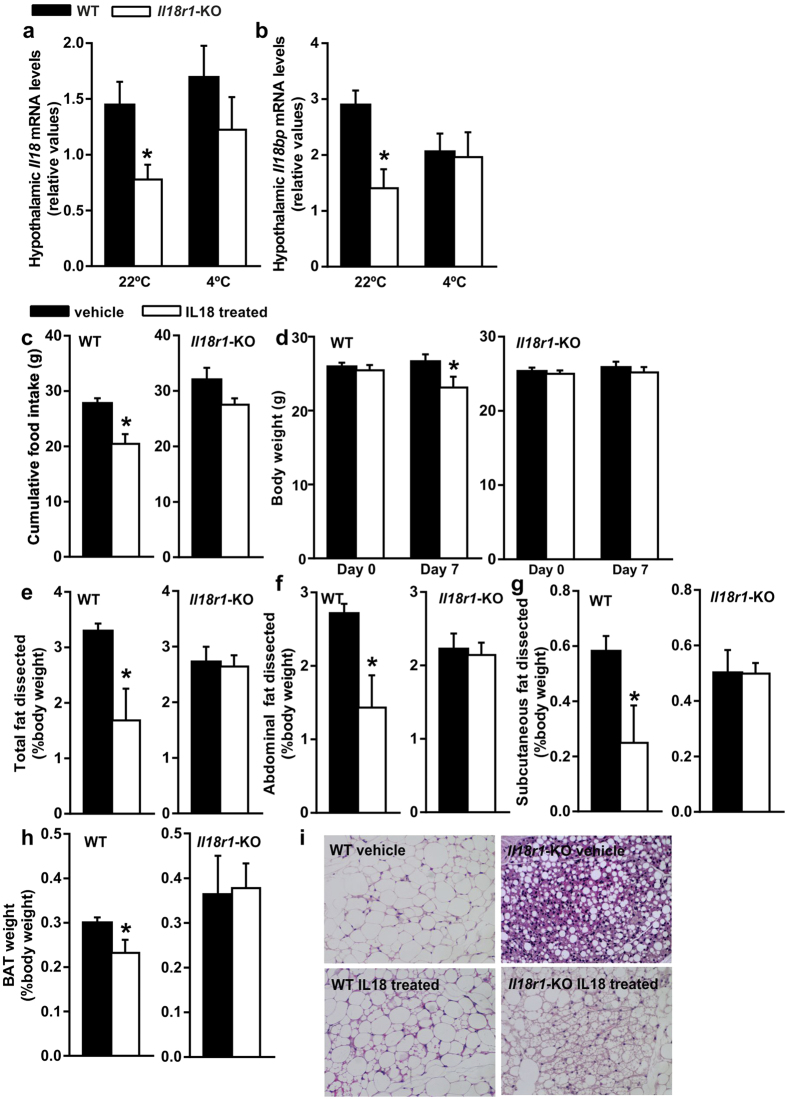
Chronic central IL18 administration induces BAT-like morphology in iWAT from *Il18r1*-KO mice. (**a**) Hypothalamic *Il18* and (**b**) *Il18bp* mRNA levels in WT and *Il18r1*-KO mice maintained at room temperature and after 4 h of transfer to a cold room (n = 6–13). (**c**) Cumulative food intake, (**d**) body weight and percentage of (**e**) total, (**f**) abdominal and (**g**) subcutaneous fat mass, and (**h**) BAT weight in relation to body weight of WT and *Il18r1*-KO mice i.c.v. treated with either saline or recombinant IL18 (20 μg/7days) (n = 4–6). (**i**) Hematoxylin-eosin staining of iWAT tissue (20X magnification). Data are expressed as mean ± SEM. *P < 0.05, versus respective WT control mice by two-tailed Student’s t-test.

To further explore whether centrally produced Il18 might play a role in the observed changes in adipose tissue plasticity in cold- exposed *Il18r1*-KO mice, we also analyzed the hypothalamic mRNA content of this cytokine and its binding inhibitor, *Il18bp*. Similarly reduced gene expression levels for *Il18* and *Il18bp* were found in the hypothalamus of *Il18r1* deficient mice as compared to WT controls, and this difference disappeared shortly upon cold challenge (Fig. 6a,b), whithout changes in *Il18*/*Il18bp* mRNA ratio (data not shown). Therefore, we determined whether central IL18 replacement in *Il18r1*-KO mice could resemble the effect of cold exposure in the promotion of iWAT browning. For this purpose, recombinant IL18 was chronically i.c.v. administered (20 μg/7days) to WT and *Il18r1*-KO mice. A higher IL18 dose has been previously shown to acutely reduce food intake in WT but not in *Il18r1*-KO mice^17^. In agreement with these data chronic IL18 icv infusion decreased food intake and body weight (Fig. 6c,d), along with significant reductions in total, abdominal, subcutaneous WAT and BAT content, in WT but not in *Il18r1* deficient animals (Fig. 6e–h). Subsequent histological analysis revealed, however, that central IL18 infusion induced the emergence of multi-locular brown-like adipocytes in iWAT tissue samples from *Il18r1*-KO mice (Fig. 6i).

## Discussion

We report herein that the IL18/IL18R1 system plays an important role on energy homeostasis. The primary finding is that deletion of the ligand or the receptor leads to divergent responses in BAT and iWAT in terms of beige reprogramming. Our data provides mechanistic insight regarding differential effects exerted by genetic ablation of either the ligand or its receptor.

In agreement with previous data^16^,^17^, in our study absence of *Il18* caused extreme susceptibility to dietary obesity due to increased food intake and feed efficiency, with the latter being an indirect sign of altered macronutrient utilization or decreased EE. In line with the second option, 10 weeks-consumption of HFD in *Il18*-KO mice resulted in significantly increased BAT weight, lowered BAT and iWAT *Ucp1* gene expression. A novel unexpected finding of the present work is that the lower obesogenic effect of HFD on *Il18r1*-null mice is associated with upregulated iWAT but not BAT *Ucp1* mRNA content. In addition, the subtle differences observed in overall fat mass content as well as in BAT and iWAT gross anatomy between mature obese *Il18r1* and *Il18*-KO mice raised on a LFD, also point to the existence of divergences between these knockout mice strains in the thermogenic activity of both tissues.

Our data indicate that lack of *Il18r1* in mice is associated with decreased whole-body EE and ambulatory activity as early as 8 weeks of age and this effect is blunted by HFD challenge. Such observations provide logical and complementary mechanisms^8^ for obesity development in this knockout strain. Increased energy expenditure (diet induced thermogenesis) and activity levels in the face of a HFD have been previously reported by other authors^36^,^37^. In agreement with these observations, dietary change caused an increment of EE and locomotor activity in both WT and *Il18r1*-KO mice. However, only in the case of EE this effect occurred with a higher intensity in animals lacking the alpha chain of the Il18 receptor (3.6 kcal/day vs 1.7 kcal/day), and blunted the previous differences between genotypes. Of note, Zorrilla and coworkers have recently demonstrated that the dietary obesity prone *Il18*-KO mice exhibited stable reduced whole body energy expenditure when transferred from a low to high fat diet^17^. Collectively these results are also in agreement with a divergence in diet induced thermogenesis between *Il18* and *Il18r1*-KO mice, which might justify their different susceptibility to dietary obesity.

To ascertain the specific role of BAT thermogenic capacity in the metabolic phenotype of *Il18r1* and *Il18*-KO mice, an acute 24 hours cold-exposure experiment was performed. We again found striking differences in rectal temperatures of *Il18r1* and *Il18*-KO mice at 2 and 4 hours-time points of cold-exposure, which were higher and lower than those of WT mice housed at the same temperature. Although thermographic recordings of iBAT temperature and the higher absolute BAT *Ucp1* gene expression in cold-exposed *Il18r1*-KO are in line with increased thermogenic activity of this tissue^32^, we failed to detect significant changes in those parameters in *Il18*-KO mice. Moreover, BAT activation in *Il18r1*-KO mice seems to be only a transient state, since a more prolonged cold-challenge (5 days) also failed to induce similar increases in either total *Ucp1* mRNA or protein levels.

Long term cold-challenge is also known to induce specific genetic and morphological changes in iWAT leading to a more BAT-like appearance^38^,^39^. Factors central to iWAT adaptation to cold include Prdm16, a master co-regulator critical for the commitment toward brown adipocyte lineage^40^, and its downstream target Ppargc1a, a transcriptional co-activator of nuclear receptors that is necessary for maximal induction of mitochondrial biogenesis and *Ucp1* expression^41^. *Prdm16* adipocyte-specific deletion in mice caused minimal effects on classical BAT, but markedly inhibited beige adipocyte function in iWAT following cold-exposure or β3-agonist treatment^42^. Besides dietary induced obesity, these mice developed insulin resistance specifically affecting WAT and liver as well as hepatic steatosis. Remarkably, in the present study *Prdm16* but also *Ppargc1a gene* expression was significantly decreased in the iWAT from 2-days cold-exposed *Il18*- KO mice. Lack of *Il18* also caused virtual absence of iWAT multilocular adipocytes, which was related to markedly decreased Ucp1 protein content specifically in this tissue. Of note, *adipo-Prdm16*-null mice did not show core-temperature alterations following 6 hours at 4 °C^42^, which is also in consistence with our data. Altogether the present findings demonstrate that ablation of *Il18* blocks the emergence of inducible brown adipocytes and its associated thermogenesis, and this effect could contribute to the metabolic disturbances in HFD-fed *Il18*-KO mice, previously described^2^,^16^,^17^ and confirmed herein (i.e extreme susceptibility to dietary obesity, increased basal glucose levels and hepatic steatosis). By contrast, Il18 binding receptor ablation markedly induced beige reprogramming in long-term cold-exposed mice and increased *Ucp1* gene expression, as we observed in iWAT from HFD-fed mice in correlation with lower fat mass content, glucose levels and hepatic triglyceride content.

Collectively these phenotypic divergences between *Il18* and *Il18r1* null mice suggest the existence of either i) compensatory adaptions to the lack of *Il18r1* that overcome the effects of *Il18* deletion or ii) a more complex receptor/signaling system for IL18.

Since experimental evidence does not support a role of IL37 as IL18R1 antagonist^18^,^43^, additional compensatory adaptations in response to knockout of *Il18r1* in mice might be related to inhibition of the IL37 signaling through this receptor. IL37 is only stably produced in inflammatory conditions as a negative feedback inhibitor to limit excessive inflammation damage^14^. Previous research has identified a seemingly paradoxical increase in inflammatory signaling in cells lacking the *Il18r1* chain, thus the lack of IL37 action may underlie this phenotype. Our results indicates, however, that iWAT browning but not BAT activation in *Il18r1* null mice coincides with increased mRNA expression of *Arg1*, a classsical marker of anti-inflammatory alternative macrophage polarization^34^. Recent studies have highlighted the importance of specific macrophage subsets presence in regulating BAT and iWAT function^34^,^35^,^44^. Obesity results in increased infiltration of WAT tissues by classically (M1) activated macrophages, which amplify inflammation and insulin resistance by secreting high amounts of pro-inflammatory cytokines (IL6, IL10 and Nos2)^45^,^46^. Conversely, alternative or anti-inflammatory macrophages (M2), which promote insulin sensitivity in visceral WAT by releasing IL10 and TGF-beta, are primarily recruited to BAT and iWAT in response to cold and produce cathecolamines to sustain adaptative thermogenesis^47^. Alternative activation of infiltrated macrophages is mainly governed by IL4 and IL13 signaling and maintained by IL10 but also IL6 through induction of IL4 receptor in these cells^48^. Interestingly, cold exposure-induced increases in iWAT UCP1 protein content are blunted in *Il6*-KO mice^29^ and this cytokine is a fundamental determinant in fat browning activation during human cancer cachexia^49^.

As other IL1 family members, IL37 also down-regulates inflammatory responses through intracrine signalling mechanisms independent of its cell membrane receptors^15^,^43^. Whether disparate results regarding thermogenic gene program activation in *Il18* and *Il18r1*-KO mice iWAT are related to yet unknown IL37 mouse homolog intracellular actions is another possibility worth investigating.

It is well established that IL18 mediates its biological effects in a variety of organ systems and cell types^9^ through stepwise binding to IL18R1 and IL18RAP, and subsequent juxtaposition of the intracellular toll/interleukin-1 receptor (TIR) domains of both receptors^50^. However, additional truncated variants of IL18R1 and IL18RAP, lacking this domain essential for recruitment of MYD88 adaptor protein and subsequent activation of (NF)-ΚΒ and MAPK pathways, have been reported in humans and rodents (arbitrary named type II receptors)^10^,^11^,^51^. Though their exact role is yet to be fully established, they are proposed to act as competitors of the canonical receptors in heterodimer formation thereby inhibiting IL18 actions^10^,^11^.

While direct peripheral effects of IL18 (i.e muscle specific) on lipid oxidation have been recently demonstrated^8^, several previous and present findings also suggest a central mode of action for this cytokine in controlling energy homeostasis and metabolic function. IL18R heterodimer subunits and spliced variants are found to be widely distributed across the brain^4^,^10^,^11^. Besides the hypothalamus and the amygdala, the highest level of expression are detected in the hypothalamic ventromedial and paraventricular nucleus, key centers in the control of food intake, BAT thermogenesis and iWAT browning^52^,^53^. According with this pattern of expression here we show that chronic IL18 administration (7-days) suppresses cumulative low-fat chow (LFD) intake in an Il18r1 dependent manner. These results are in agreement with a previous report that i.c.v IL18 in higher dose/dose time (1 or 2 nmol ≤40 μg, acutely administered) inhibits 24-h HFD intake in fasted WT but not in *Il18r1* deficient mice. However, our present observation that i.c.v. IL18 infusion induces a slight emergence of brown-like features in *Il18r1*-KO iWAT samples, raises the question whether the short forms of the receptor play an additional role in energy homeostasis activating browning in response HFD or cold challenge. In line with this hypothesis, we also report that lack of *Il18r1* induces compensatory down-regulations of hypothalamic *Il18* and *Il18 bp* as well as BAT *Il18* gene expression, and acute cold challenge blunts these effects. Given the reported low levels of CNS expression of *Il18r* short variants^9^,^10^,^11^, additional transgenic mice with conditional and tissue specific deletion of these receptor isoforms would be helpful to decipher the exact site and mechanisms of action.

In summary our data show that lack of *Il18* but not *Il18r1* in mice led to an increased susceptibility to dietary obesity. Detailed analysis of the different compartments involved in energy homeostasis indicate that the disparate effects exerted by genetic ablation of the ligand and the receptor are likely mediated by divergent responses to thermogenic stimuli in BAT as well as the capacity of subcutaneous AT browning. Understanding of the molecular mechanisms by which ablation of IL18R1 exerts its effects may provide a novel therapeutic approach to treat obesity.

## Material and Methods

### Ethics statement

Animal experimental procedures were conducted according to the regulations of the European Communities directive of September 2010 (2010/63/ECC) and Spanish legislation (BOE 34/11370–421, 2013) regulating animal research. All the research procedures included in this study were approved by University of Santiago de Compostela Bio-ethics Committee.

### Animals

8-weeks old *Il18* and *Il18r1* deficient mice (B6.129P2–Il18 < tm1Aki >/J and B6.129P2–Il18r1 < tm1Aki >/J) and their congenic controls C57BL/6J mice (WT), were used in all experiments. For the diet intervention and cold exposure studies animals were initially bred on site from homozygous pairs purchased from The Jackson Laboratory (Charles River Laboratories, Barcelona, Spain; stock numbers 004130, 004131 and 000664, respectively), and housed under 22°C temperature and 12 hours light-dark conditions. Subsequently, for cold-challenge confirmatory experiments, central IL18 infusion, and hypothalamic *Il18* and *Il18bp* mRNA assays we used heterozygous *Il18r1*-KO and WT littermate mice as well as in *Il18r1* deficient and WT animals generated from homozygous breeding pairs, which were F1 offspring from *Il18r1*-KO heterozygote breeders^54^.

### Diet intervention

Mice were fed either standard rodent chow (CH: 13% fat, 67% carbohydrate and 20% protein on a caloric basis; 2014S Harlan Teklad, Barcelona, Spain) or high fat diet (HFD: 60% fat, 20% carbohydrates and 20% protein on a caloric basis; D12492, Research Diets, New Brunswick, NJ, USA) for 10 weeks. Body weight and food intake were measured weekly in 4–6 cages per treatment group (2–3 animals/cage). At the end of the study period whole-fat and lean mass content was measured by NMR spectroscopy (EchoMRI–700™, Echo Medical Systems, Houston, TX, USA). Animals were sacrificed by decapitation under ketamine-xylazine anesthesia for blood collection and, fat depots dissection and weighing^55^.

For complementary body weight and body composition analysis a third group of WT, *Il18* and *Il18r1*-KO mice (5–6 mice/genotype) fed standard rodent chow (2019s, Teklad Global, Harlan, Spain) were used. At 24 weeks of age and prior sacrifice, body length was measured with a ruler from nose to anus on sedated animals. iWAT and BAT samples were removed quickly and fixed in 4% paraformaldehyde for subsequent histological analysis.

### HFD challenge, energy expenditure and locomotor activity measurements

Analysis of energy metabolism by indirect calorimetry was performed in independent groups of WT and *Il18r1*-KO mice (6 mice/genotype). After 2 days of acclimation, mice were individually monitored in a TSE LabMaster system (TSE Systems, Bad Homburg, Germany) as previously described^55^. Data on gas exchanges, locomotor activity, and food intake were collected every 20 min for two consecutive 48 h-periods of feeding chow and HFD. Activity monitoring was performed simultaneously with infrared sensor pairs arranged in strips for horizontal (X level) and vertical (Z level) activity. Respiratory exchange ratio (volume of CO2 produced per volume of O2 consumed (ml/kg of body weight/ per h), and energy expenditure were also calculated for the light and dark phases.

### Cold-challenge

For short- and long term-cold-challenge experiments individually housed mice with free-access to standard chow diet were exposed to a 4 °C ambient temperature for 1, 4 or 24 hours, and 2 or 5 days. Rectal or surface temperatures were collected at indicated times using a digital thermometer (RS, Barcelona, Spain) or an infrared thermographic camera (Therma E40; FLIR, Croissy-Beaubourg, France). Animals (8–12 mice/genotype) were acclimated to individual cages and rectal probing during three days. As infrared radiations are blocked by Plexiglas or stainless steel, one day before the thermographic recordings start, mice (5–8 mice/genotype) were lightly anesthetized with ketamine–xylacine and shaved between the scapulae and over the lumbar back region, to expose the skin for temperature readings in these areas (TiScap and TBack, respectively)^33^. Each infrared digital image simultaneously captured TiScap and TBack, and was analyzed with a specific software package (FLIR Tools Software). The temperature surrounding interscapular and lumbar back region for one particular animal was calculated as the average temperature in defined areas recorded by analyzing those pictures^32^. After completion of the studies, mice were sacrificed and, iWAT and BAT samples for histological or expression studies were dissected out, frozen on dry-ice and stored at –80°C.

### Intracerebroventricular infusions

To assess the long-term central effects of the IL18 system on food intake, body composition and iWAT morphology, 10–11 weeks old *Il18r1*-KO and WT mice (4–7 mice/genotype/treatment) were infused ICV with saline or recombinant IL18 (20 μg/mouse/7 days, Sino Biological, Inc., Beijing, China)^56^. For this purpose, brain infusion canulae were placed stereotaxically into the lateral ventricle as previously described^56^. A catheter tube was connected from the brain infusion cannula to the osmotic minipump (model 1007D, total volume 100 μL, flow rate 0.5 μL/h; Alzet Osmotic Pumps, Durect, CA, USA) flow moderator.

### Hematoxylin/eosin staining

iWAT and BAT samples were fixed 24 hour in 10% formalin buffer and then were dehydrated and embedded in paraffin by a standard procedure. Sections of 3 μm were made in a microtome and staining in a standard Hematoxylin/Eosin Alcoholic (BioOptica, Milan, Italy) procedure as manufacture instructions.

### Oil Red Staining

Frozen 8 μm-sections of the livers were cut on a cryostat and stained in filtered Oil Red O for 10 minutes. Sections were washed in distilled water and counterstained with Mayers hematoxylin for 3 minutes. Sections were mounted in aqueous mountant (glycerin jelly).

### Levels of Serum Metabolites and Hormones

Serum glucose and triglyceride levels were assessed using commercial kits based on a enzymatic colorimetric method (Glucose-TR and Triglycerides-LQ, Spinreact, Girona, Spain). Serum leptin and adiponectin levels were measured using enzyme-linked immunosorbent assay kits (Merck Millipore, Madrid, Spain) following the manufacturer’s instructions.

### Liver Triglyceride content

Liver samples (40–50 mg) were extracted in 1 ml isopropanol. After centrifugation at 4 °C at 10000 rpm for 15 min, 10-μl aliquots of supernatant were added to 200 μl reagent (Triglycerides-LQ, Spinreact, Girona Spain) for enzymatic colorimetric determination of triglyceride concentration.

### Real time qPCR

Total RNA was extracted from frozen iWAT and BAT samples using TRIzol according to the manufacturer’s instructions (Invitrogen, Barcelona, Spain). Reverse transcription and qPCR (TaqMan®, Applied Biosystems, Barcelona, Spain) were performed in duplicates as described earlier^55^. Complementary DNAs were synthesized from 500 ng of total RNA in a 30 μl reaction using 200 U Maloney murine leukemia virus reverse transcriptase and random hexamer primers (Invitrogen, Barcelona, Spain). Quantitative real-time PCR was performed using an ABI PRISM 7300 HT Sequence Detection System (Applied Biosystems; Foster City, CA, USA) with 2 μl aliquots of the resulting cDNAs and specific Taqman qRT-PCR primers and probes (Supplementary Table 2). Amplification of *18S* rRNA (*Rn18S*) was performed at the same time to normalize the level of mRNA expression. A non template reaction was included during each experiment to control for DNA contamination. The PCR cycling conditions included an initial denaturation at 50 °C for 10 min followed by 40 cycles at 95 °C for 15 s and 60 °C for 1 min. A standard curve was run in each assay, with an arbitrary value assigned to the highest standard and corresponding values to the subsequent dilutions. Each cDNA sample was run in duplicate. The relative abundance each gene targets were normalized against that of *Rn18s.* The total or absolute levels of *Ucp1* mRNA were calculated by multiplying the specific *Ucp1* value per μg of RNA with the total amount of extracted RNA as previously described^25^.

### Western Blot

Western blots were performed as previously described. Briefly, total protein lysates from iWAT and BAT (180 and 30 μg) samples were separated by SDS-PAGE, electrotransferred onto a polyvinylidene difluoride membrane (Immobilon-P, Millipore, Billerica, MA, USA) and probed with antibodies against Ucp1 (ab10983, Abcam, Cambridge, United Kingdom), and alpha-tubulin (T5168, Sigma-Aldrich, St. Louis, MO, USA). The antibodies dilution was 1:1000. For protein detection biotinylated secondary antibody conjugates, diluted (1:5000) and chemiluminescence (Thermo Fisher Scientific, Madrid, Spain) were used. Then, the membranes were exposed to radiograph film (Super RX, Fuji Medical X-Ray Film; Fujifilm, Tokyo, Japan) and developed with developer and fixing liquids (AGFA, Mortsel, Belgium) under appropriate dark room conditions. Protein levels were relatively quantitated by normalization with alpha-tubulin levels in the same lysate. The total or absolute protein levels were calculated by multiplying the specific Ucp1 protein level per mg homogenate protein with the total amount of homogenate protein as previously described^25^.

### Statistical analysis

Data were analyzed using Graph Pad Prism version 6.00 for Windows (GraphPad Software, La Jolla, California, USA) and given as means ± SEM. Statistical significance was determined by two-tailed Student’s t test and one- or two-way analysis of variance (ANOVA), followed by Bonferroni post-hoc tests.

## Additional Information

**How to cite this article**: Pazos, P. *et al.* Divergent responses to thermogenic stimuli in BAT and subcutaneous adipose tissue from *interleukin 18* and *interleukin 18 receptor 1*-deficient mice. *Sci. Rep.* **5**, 17977; doi: 10.1038/srep17977 (2015).

## Supplementary Material

Supplementary Information

## Figures and Tables

**Table 1:** Serum parameters in *Il18r1* and *Il18*-KO mice after ten weeks of HFD.

	WT	*Il18r1*-KO	*Il18*-KO
Leptin (ng/ml)	22.75 ± 1.16	25.80 ± 5.81	45.76 ± 4.82*
Adiponectin (μg/ml)	13.83 ± 1.16	12.39 ± 10.81	18.36 ± 14.25*
Glucose (mmol/L)	10.91 ± 1.11	12.04 ± 0.57	14.68 ± 0.98*
Triglycerides (mmol/L)	0.89 ± 0.04	0.99 ± 0.06	0.37 ± 0.03***

Values represent means ± SEM. n = 8–13. *P < 0.05 and ***P < 0.001 versus respective WT mice fed the same diet by one-way ANOVA.
