# Supplementary material for: Divergent responses to thermogenic stimuli in BAT and subcutaneous adipose tissue from interleukin 18 and interleukin 18 receptor 1-deficient mice
Source: Sci Rep. 2015 Dec 10;5:17977. doi: 10.1038/srep17977 (PMC4674707; doi:10.1038/srep17977)
Supplement: Supplementary Information [file srep17977-s1.pdf]

Supplementary information

Full title: Divergent responses to thermogenic stimuli in BAT and subcutaneous adipose tissue from *interleukin 18* and *interleukin 18 receptor 1*-deficient mice

Authors: Patricia Pazos[1,2,3], Luis Lima[1,2], Sulay Tovar[1,2,3], David Gonzalez-Touceda[1,2,3], Carlos Diéguez[1,2,3], María C. García*[1,2,3].

Affiliations: [1] Department of Physiology/Research Center of Molecular Medicine and Chronic Diseases (CIMUS). University of Santiago de Compostela and [2] Instituto de Investigación Sanitaria de Santiago de Compostela, Santiago de Compostela, Spain. [3] CIBER Fisiopatología Obesidad y Nutrición (CB06/03), Instituto de Salud Carlos III (ISCIII, Ministerio de Economía y Competitividad (MINECO), Spain).

* **Correspondence:** Maria C. García. Department of Physiology-CIMUS, Avenida de Barcelona s/n, 15782 Santiago de Compostela, Spain. Phone: (+34) 881 815431. Fax: (+34) 881 812432. E-Mail: maria.garcia.garcia@usc.es

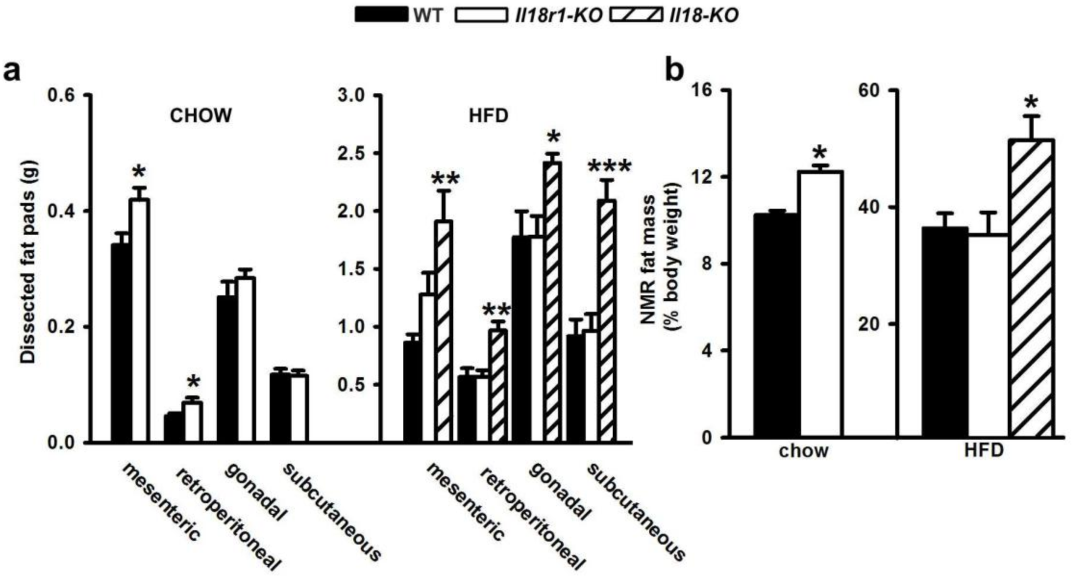

Supplementary Figure 1: Divergent responses to dietary obesity in *Il18r1* and *Il18*-KO mice

(a) Absolute dissected abdominal (mesenteric, retroperitoneal and gonadal) and subcutaneous fat mass of WT, *Il18r1* and *Il18*-KO mice fed a regular chow (CHOW, left panel) or a 60% high-fat diet (HFD, right panel) from 8 to 18 weeks of age (n=8-13). (b) Relative fat body mass determined by NMR at the end of the study calculated in percentage to WT body weight (n=8-13). Data are expressed as mean ±SEM. *P<0.05 and **P<0.01 versus respective WT mice fed the same diet by two-tailed Student´s t-test and one way ANOVA.

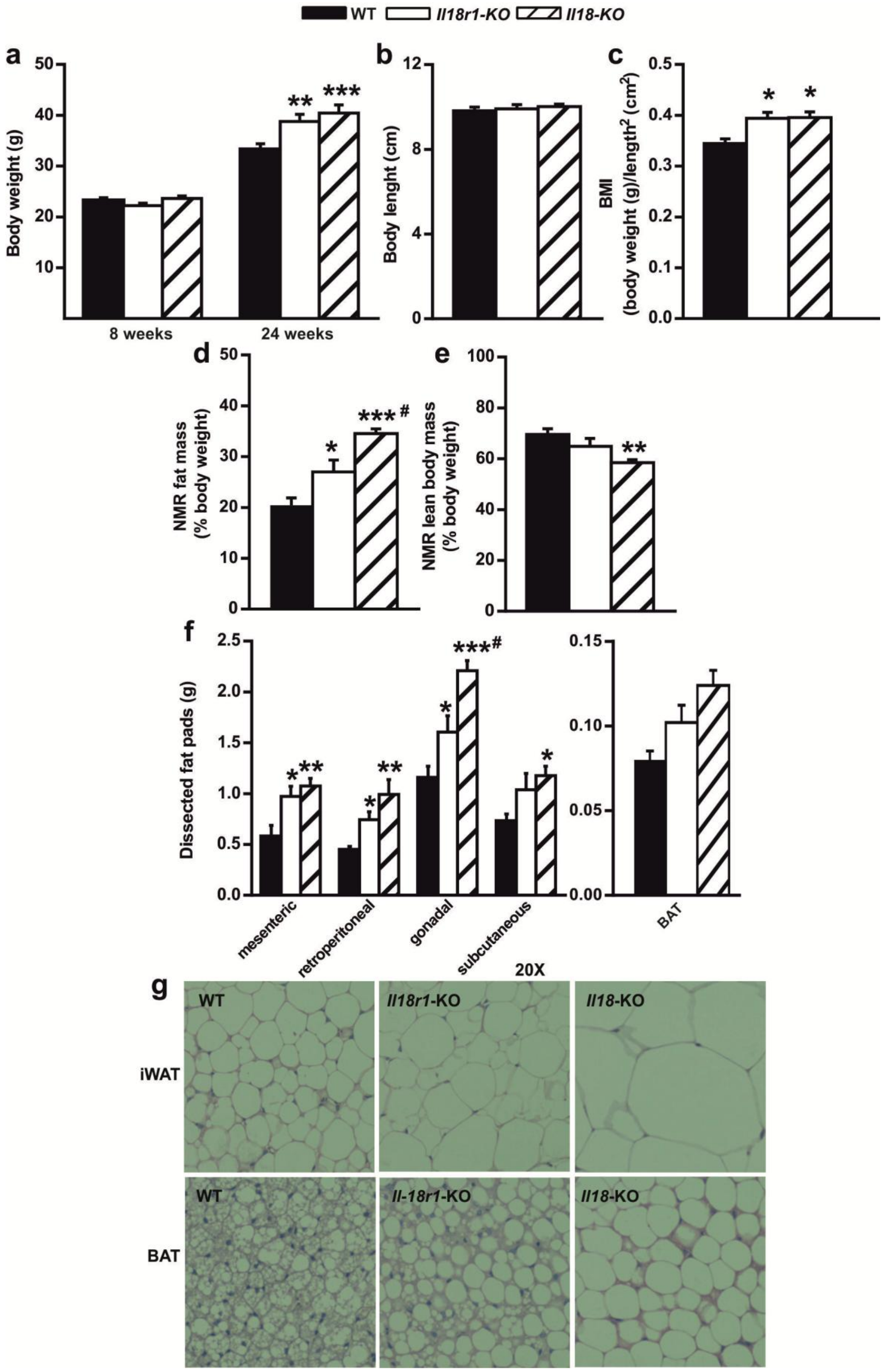

WT
Il18r1-KO
Il18-KO
a
Body weight (g)
8 weeks
24 weeks
**
***
b
Body lenght (cm)
c
BMI
(body weight (g)/length^2 (cm^2))
*
d
NMR fat mass
(% body weight)
***#
e
NMR lean body mass
(% body weight)
f
Dissected fat pads (g)
mesenteric
retroperitoneal
gonadal
subcutaneous
BAT
g
20X
iWAT
BAT
Il-18r1-KO

Supplementary Figure. 2: *Il18r1* and *Il18*-KO mice develop mature-onset obesity

(a) Body weight of WT, *Il18r1* and *Il18*-KO mice at 8 and 24 weeks of age (n=5-6). (b) Body length,(c) body mass index (BMI), (d) NMR fat mass, (e) NMR lean body mass (both calculated as percentage of body weight), and (f) weight of dissected fat pads in WT, *Il18r1* and *Il18*-KO mice at 24 weeks of age. (g) Hematoxylin-eosin staining of iWAT and BAT tissue (20X magnification). Data are expressed as mean ±SEM. *P<0.05, **P<0.01 and ***P<0.001 versus respective WT control mice and # P<0.05 versus *Il18r1*-KO mice by one way ANOVA or two way ANOVA for repeated measurements. ((a) genotype F (2, 13) = 4.886, time F (1, 13) = 273.5 and genotype X time interaction F (2, 13) = 16.49 ; P<0.05 and P<0.0001)

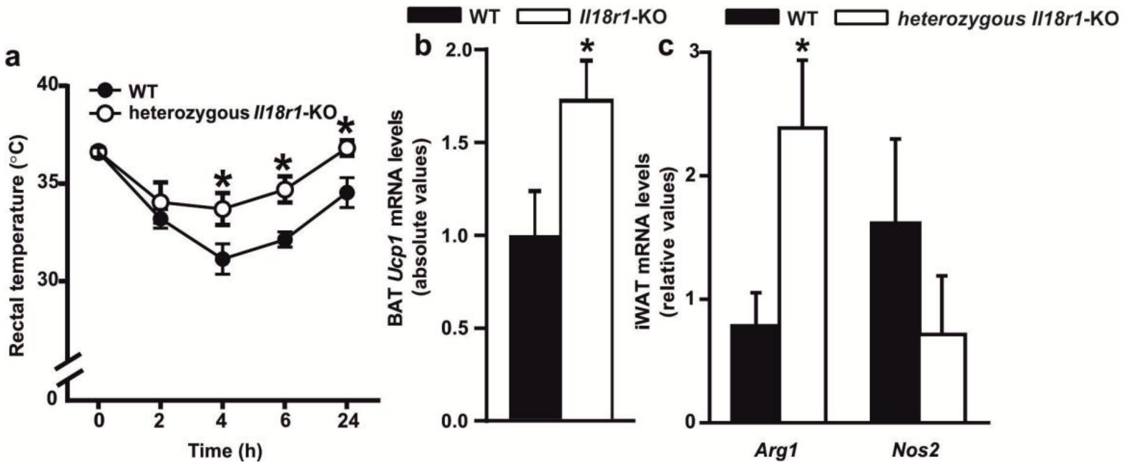

Supplementary Figure 3: Breeding scheme effect on rectal temperature, BAT and iWAT responses to short-term cold exposure

(a) Rectal temperature, (b) BAT *Ucp1* and, (c) alternative and classic macrophage polarization markers (*Arg1* and *Nos2*) mRNA levels were measured in either littermate WT and heterozygous *Il18r1*-KO mice (n=5) or WT and *Il18r1*-KO mice generated from homozygous mating of F1 offspring from *Il18r1*-KO heterozygote breeders (n=5-6). Data are expressed as mean ±SEM. *P<0.05 versus respective WT control mice t-test or two way ANOVA for repeated measurements. ((a) genotype F (4, 32) = 18.76, time F

(1, 8) = 9.696 and genotype X time interaction F (4, 32) = 1.994; $P<0.05$, $P<0.0001$ and $P = 0.119$)

Supplementary Table 1: BAT weights of mice used for protein and RNA expression studies

**Protein studies**	**Temperature, time**	**WT**	***Il18r1*-KO**	***Il18*-KO**
	4ºC, 5 days	0.120±0.008	0.147±0.011	0.127±0.012
**RNA studies**	**Diet**	**WT**	***Il18r1*-KO**	***Il18*-KO**
	chow	0.084±0.012	0.097±0.007	ND
	HFD	0.129±0.010	0.145.0±0.006	0.167±0.014*
	**Temperature, time**	**WT**	***Il18r1*-KO**	***Il18*-KO**
	22ºC	0.071±0.011	0.090±0.006	0.082±0.008
	4ºC, 4h	0.104±0.018	0.092±0.014	0.074±0.009
	4ºC, 2 days	0.126±0.014	0.122±0.018	0.114±0.028
	4ºC, 5 days	0.138±0.015	0.152±0.012	0.122±0.009

[1]Values represent means± SEM. n=5-7. * $P<0.05$ versus respective WT mice fed the same diet by one-way ANOVA.

Supplementary Table 2: Primers and probes used for real-time PCR

Gene	Gene bank	*Primers and* probes sequences[a]	Product size (bp)	Modified from
Rn18s	NR_003278.1	F:5´CGCCGCTAGAGGTGAAATTC3´<br>R:5´CGAACCTCCGACTTTCGTTCT3´<br>Pb:5´CCGGCGCAAGACGGACCAGA3´	101	[1]
Ucp1	NM_009463.2	F:5´ GCAGATATCATCACCTTCCCG 3<br>R:5´ CCTGGCCTTCACCTTGGAT,3´<br>Pb:5´ AACGCCTGCCTCTTTGGGAAGCAA3´	66	[2]
Il18	Mm00434225_m1	Gene Expression Assay Applied Biosystems	104	
Il18bp	Mm01274147_g1	Gene Expression Assay Applied Biosystems	59	
Arg1	Mm00475988_m1	Gene Expression Assay Applied Biosystems	65	
Nos2	Mm00440502_m1	Gene Expression Assay Applied Biosystems	66	
Ppargc1a	NM_008904.1	F:5´ TTTTTGGTGAAATTGAGGAATGC 3<br>R:5´ CGGTAGGTGATGAAACCATAGCT- 3<br>Pb:5´ GTCTCCATCATCCCGCAGATTTACGG 3	73	[3]
Prdm16	Mm00712556_m1	Gene Expression Assay Applied Biosystems	72	

[a]F, Forward; R, reverse; Pb, probe

References

1 Capurso, G. *et al.* Gene expression profiles of progressive pancreatic endocrine tumours and their liver metastases reveal potential novel markers and therapeutic targets. *Endocr Relat Cancer* **13**, 541-558, doi:10.1677/erc.1.01153 (2006).

2 Mashiko, S. *et al.* Characterization of neuropeptide Y (NPY) Y5 receptor-mediated obesity in mice: chronic intracerebroventricular infusion of D-Trp(34)NPY. *Endocrinology* **144**, 1793-1801, doi:10.1210/en.2002-0119 (2003).

3 Heijboer, A. C. *et al.* Sixteen hours of fasting differentially affects hepatic and muscle insulin sensitivity in mice. *J Lipid Res* **46**, 582-588, doi:10.1194/jlr.M400440-JLR200 (2005).
